# Experimental investigation of a combinational iron chelating protoporphyrin IX prodrug for fluorescence detection and photodynamic therapy

**DOI:** 10.1007/s10103-021-03367-1

**Published:** 2021-07-04

**Authors:** Anette Magnussen, Charlotte Reburn, Alexis Perry, Mark Wood, Alison Curnow

**Affiliations:** 1 European Centre for Environment and Human Health, Environment and Sustainability Institute, University of Exeter Medical School, University of Exeter, Penryn Campus, Cornwall, TR10 9FE UK; 2 Nuffield Department of Surgical Sciences, John Radcliffe Hospital, University of Oxford, Oxford, OX3 9DU UK; 3 Biosciences, College of Life and Environmental Sciences, University of Exeter, Geoffrey Pope Building, Stocker Road, Exeter, EX4 4QD Devon UK

**Keywords:** Fluorescence, Iron chelation, Iron chelating agent, Photodetection, Photodynamic therapy (PDT), Protoporphyrin IX (PpIX)

## Abstract

Photodynamic therapy (PDT) is an oxygen-dependent, light-activated, and locally destructive drug treatment of cancer. Protoporphyrin IX (PpIX)-induced PDT exploits cancer cells’ own innate heme biosynthesis to hyper-accumulate the naturally fluorescent and photoactive precursor to heme, PpIX. This occurs as a result of administering heme precursors (e.g., aminolevulinic acid; ALA) because the final step of the pathway (the insertion of ferrous iron into PpIX by ferrochelatase to form heme) is relatively slow. Separate administration of an iron chelating agent has previously been demonstrated to significantly improve dermatological PpIX-PDT by further limiting heme production. A newly synthesized combinational iron chelating PpIX prodrug (AP2-18) has been assessed experimentally in cultured primary human cells of bladder and dermatological origin, as an alternative photosensitizing agent to ALA or its methyl or hexyl esters (MAL and HAL respectively) for photodetection/PDT. Findings indicated that the technique of iron chelation (either through the separate administration of the established hydroxypyridinone iron chelator CP94 or the just as effective combined AP2-18) did not enhance either PpIX fluorescence or PDT-induced (neutral red assessed) cell death in human primary normal and malignant bladder cells. However, 500 µM AP2-18 significantly increased PpIX accumulation and produced a trend of increased cell death within epithelial squamous carcinoma cells. PpIX accumulation destabilized the actin cytoskeleton in bladder cancer cells prior to PDT and resulted in caspase-3 cleavage/early apoptosis afterwards. AP2-18 iron chelation should continue to be investigated for the enhancement of dermatological PpIX-PDT applications but not bladder photodetection/PDT.

## Introduction

Photodynamic therapy (PDT) is based on the activation of a photosensitizer by visible light and the transfer of energy to molecular oxygen, creating reactive oxygen species (ROS) that induce oxidative stress and thus cell death [1]. 5-Aminolevulinic acid (ALA)-induced-PDT exploits the cell’s own innate heme biosynthesis pathway to generate the natural endogenous photosensitizer protoporphyrin IX (PpIX; the precursor to heme). Free heme inhibits further ALA synthesis, and this natural regulation step can be bypassed by purposefully adding exogenous ALA, which then drives the pathway to operate at maximal capacity. Cellular accumulation of PpIX occurs as the final step (the insertion of ferrous iron (Fe^2+^) into PpIX by ferrocheletase to form heme) is relatively slow [1]. This process occurs at a faster rate in malignant cells (which generally have upregulated and less well controlled heme biosynthesis) than in normal cells and so a temporally selective window occurs when red light (635 nm) can be applied to activate PpIX within the tumor, while sparing surrounding normal cells [1].

Within dermatology, a topical cream containing the small, soluble precursor to PpIX (ALA or its methyl ester, methyl-aminolevulinate (MAL; Metvix, Galderma, UK)) is applied to the area of skin to be treated [2]. Although effective treatment outcomes associated with excellent cosmesis have been demonstrated in licensed lesions (actinic keratosis, Bowen’s disease and superficial basal cell carcinoma (BCC)) when the disease remains superficial [3–5] or alternatively in mild to moderate inflammatory acne [6], efforts continue to increase the efficacy and extend the applications of topical PpIX-PDT particularly to treat thicker nodular BCC or acrally located conditions [7]. Poor penetration into deeper layers can be improved clinically by employing more lipophilic ALA derivatives (e.g., MAL) [8–10] or nano-emulsion formulations (e.g., ALA; Ameluz, Spirit Healthcare, UK) [5] and by performing skin pre-treatments [11–13]. There is also interest in applying PDT beyond dermatology, in a wide range of oncological/non-oncological applications [14].

PpIX’s natural fluorescence can also be employed for photodetection of bladder cancer [15–17] or fluorescence-guided surgical resection of high-grade glioma of the brain using orally administered ALA [18], as PpIX exhibits characteristic red fluorescence (at 635 nm and 700 nm) when excited by blue light (410 nm). Cells that have accumulated PpIX can therefore be identified through non-invasive fluorescence monitoring [19]. The more lipophilic hexyl ester derivative, HAL (Hexvix, Photocure, Norway) [20] doubled PpIX accumulation in a human bladder cancer photodetection pilot study, with PpIX being evenly distributed throughout the urothelial layers [15, 21]. A subsequent clinical study of HAL for urothelial cell carcinoma also demonstrated promising results [22], and photodetection of bladder cancer with HAL now has FDA and European approval [23]. Although this technique has increased tumor detection, the long-term impact on recurrence rates still remains unclear [24].

**Scheme 1:**
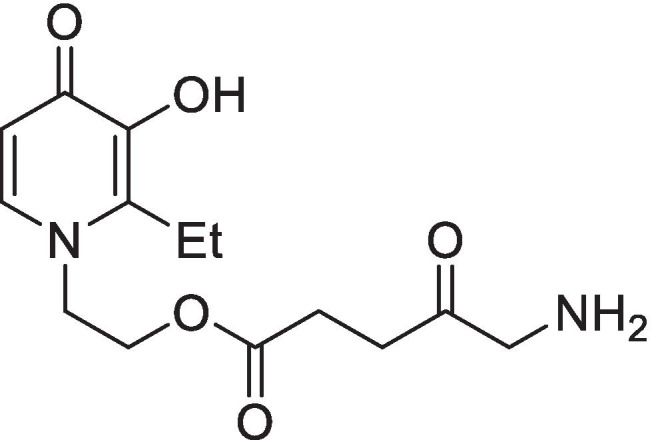
The molecular structure of the hydroxypyridinone iron chelating PpIX prodrug, AP2-18

Using an iron chelating agent to reduce intracellular iron can inhibit the conversion of PpIX to heme, resulting in greater PpIX accumulation. ALA and hydroxypyridinone iron chelating agent co-administration has been previously demonstrated to successfully increase PpIX-PDT efficacy in vitro, in vivo, and during clinical application [25–28]. 1,2-diethyl-3-hydroxypyridine-4-one (CP94) has been demonstrated to be the best iron chelator for this application as it diffuses through the cell membrane better than other clinically available iron chelators and has a greater selectively for iron than other transition metals [29, 30]. A novel-combined conjugate, AP2-18, which links ALA to CP94 via a cleavable ester (Scheme 1) has been derived [31] to overcome co-administration issues by releasing the two agents simultaneously following cellular uptake and ubiquitous cytosolic esterase release [32, 33]. Promising initial experimental findings of AP2-18 activity within human normal skin fibroblasts and skin cancer cells [34, 35] have been extended here as well as investigated within human cells of bladder origin for the first time to assess the feasibility of AP2-18 as an alternative to ALA/MAL administration in skin or HAL application in bladder for fluorescence photodetection and/or PDT treatment.

## Materials and methods

Normal human primary bladder epithelial cells and two types of human urinary bladder transitional cell carcinoma (RT-4, from a well differentiated tumor stage T2/grade G1 and aggressive RT-112, from a tumor grade G2) were cultured aseptically in 96-well plates for experimentation (primary bladder cells and RT-4, ATTC, Germany; RT-112, ECACC, UK). Additional experimentation was conducted with human primary epithelial squamous carcinoma cells (A431, ECACC, UK).

**Fig. 1:**
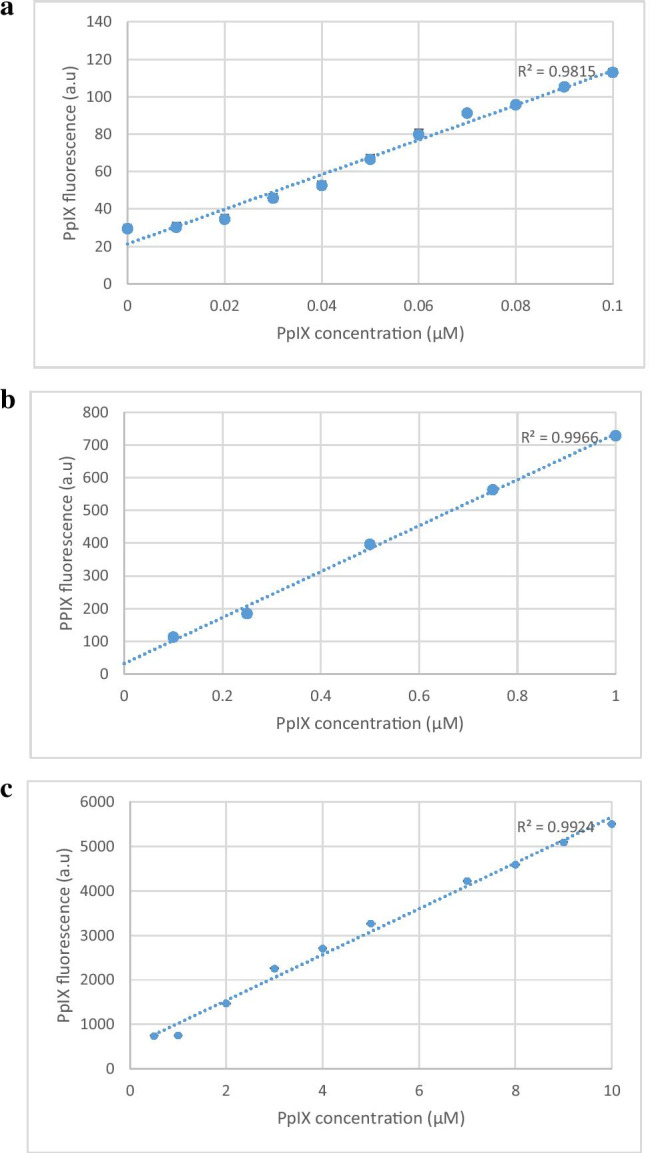

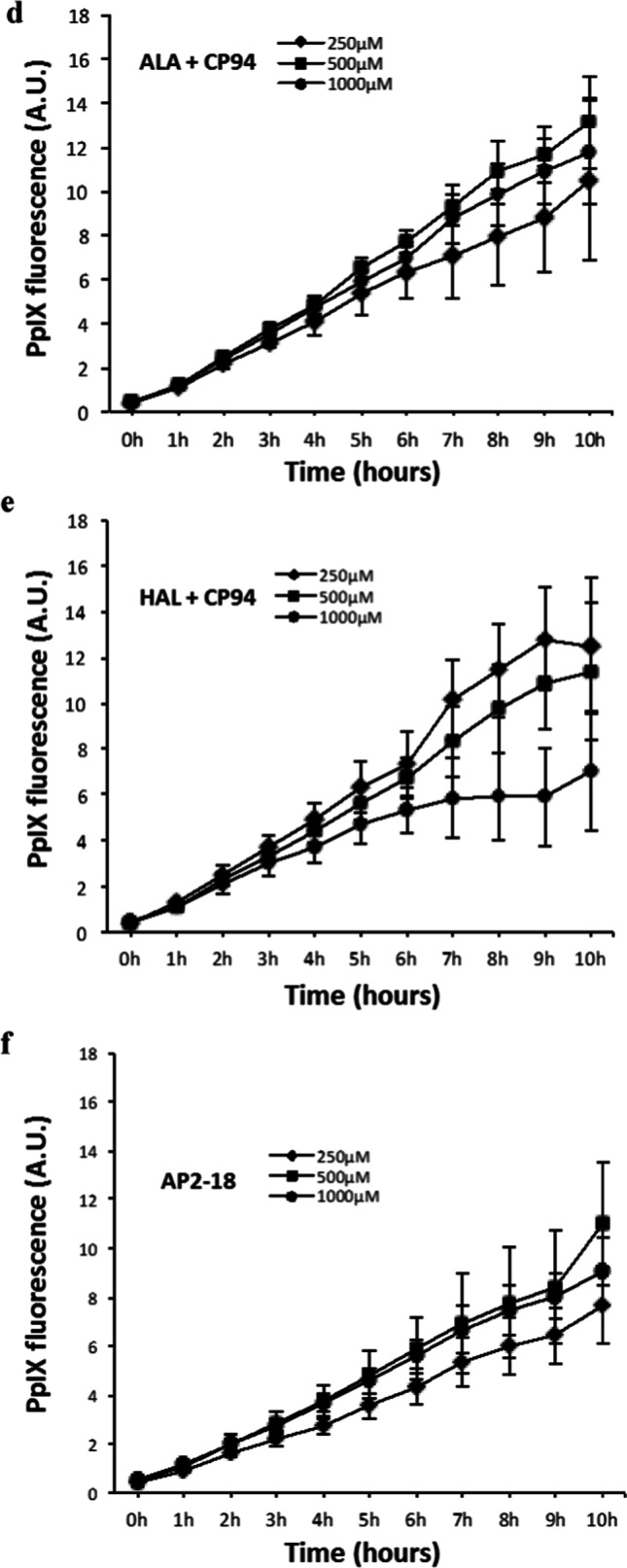

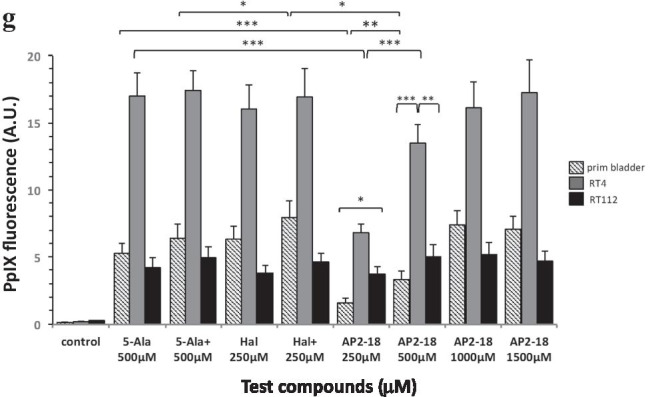
Panels **a**–**c** demonstrate the linear relationship between varying amounts of synthetic PpIX (up to 0.1, 1.0, and 10.0 µM PpIX respectively) and fluorescence intensity (in arbitrary units) observed during preliminary experimentation. Panels **d**–**f** record PpIX fluorescence accumulation measured in hourly intervals over 10 h in RT-112 bladder cancer cells (n = 5). RT-112 cells were treated with increasing concentrations of **d** ALA + CP94; **e** HAL + CP94 and **f** the composite prodrug AP2-18. CP94 was added in equimolar concentrations. Statistical comparison (Welch two sample t-test) of fluorescence intensities between each individual treatment group at 6 h and 10 h produced p-values > 0.05. Bars indicate the standard error of the mean. Panel **g** illustrates mean PpIX fluorescence measured following 6-h incubation in normal primary bladder cells and RT-4 & RT-112 bladder cancer cells (n = 5). The cells were treated with ALA alone (5-Ala), ALA + CP94 (5-Ala +), HAL alone (Hal), HAL + CP94 (Hal +), or the composite drug AP2-18 in increasing concentrations (250–1500 µM). CP94 was added in equimolar concentrations. Statistical comparison between individual experiments (Welch two-sample t-test) confirmed that PpIX accumulation in RT-4 cells was significantly higher than in RT-112 and primary bladder cells in all experiments with p-values ≤ 0.001. The statistical significances within the experimental groups are otherwise indicated as *p ≤ 0.05, **p ≤ 0.005 and ***p ≤ 0.001 respectively. Bars indicate the standard error of the mean

Initial investigations tested AP2-18 against the clinically approved prodrugs ALA and HAL (250, 500 or 1000 µM) ± the hydroxypyridinone iron-chelator, CP94. Fluorescence intensity (excitation λ = 405 ± 12 nm; detection λ = 635 ± 12 nm) was measured hourly (in arbitrary units) with a Bio-tek Synergy HT plate reader (Swindon, UK) as an indication of PpIX in RT-112 cells (n = 5) over 10 h at 37˚C following the generation of standard curves (Fig. 1a–c) to demonstrate that fluorescence was linearly aligned to synthetic PpIX (Sigma, UK) concentration. Further PpIX fluorescence experimentation utilized up to 1500 µM AP2-18 and monitored all three bladder cell types (n = 5) as well as skin cancer cells up to 6 h (a minimum of in triplicate on three separate occasions) with MAL replacing HAL in this cell type as MAL is utilized clinically within dermatology (unlike HAL). Background low light intensity conditions were employed in the laboratory as a precaution throughout all experimentation to limit potential photosensitizer photobleaching.

Photodynamic effects were investigated within all cell types after 6-h incubation with the respective prodrugs, to match earlier investigations and because PpIX accumulation observed appeared consistent up to this time point. Preliminary experimentation established that 500 µM ALA and 250 µM HAL were most effective in the bladder cell types. These concentrations were therefore adopted for continuity and because higher concentrations did not significantly increase RT-112 PpIX accumulation. For the evaluation of the new composite drug AP2-18, 250–1500 µM was employed in the three bladder cell types and ≤ 1500 µM in the skin cancer cells using a clinical Aktilite LED array (Galderma, UK) as the PDT light source. Bladder cells (n = 5) were irradiated 6 h after treatment (λ = 635 nm; dose = 30Jcm^−2^) and cell viability/death assessed 16 h later. The skin cancer cells were treated similarly except the clinical dermatology light dose of 37Jcm^−2^ was employed with 500 µM MAL and 250 µM ALA (because these concentrations previously produced effective results in this cell type). Appropriate dark (drug only), blank (no drug), and positive (lysed) control groups were also conducted in parallel to the test groups.

Sixteen hours after PDT, the 96-well plates were centrifuged (900 rpm; 3 min), and the adherent cells utilized for the neutral red assay. As viable cells take up neutral red, the dye can be extracted, and its color intensity utilized to indicate how many survived [36]. A 7.5-µl neutral red solution was added to each well for 2 h. The supernatant was removed; the cells washed twice with phosphate buffered saline (PBS), and the neutral red dissolved with 200 µl solubility buffer (49 parts H_2_O, 50 parts 99.9% ethanol and one part glacial acid). Absorbance as a measurement of cell viability was recorded at 540 nm. The number of surviving cells was presented in reference to the untreated positive control, which was set to 100%. The increase in cell death after PDT in comparison to the control cells was in all cases significantly higher, which confirmed the cytotoxic effect of PDT. Statistical analysis was conducted utilizing the Welch two sample t-test (within Microsoft Excel), with p-values < 0.05 being considered significant.

**Fig. 2:**
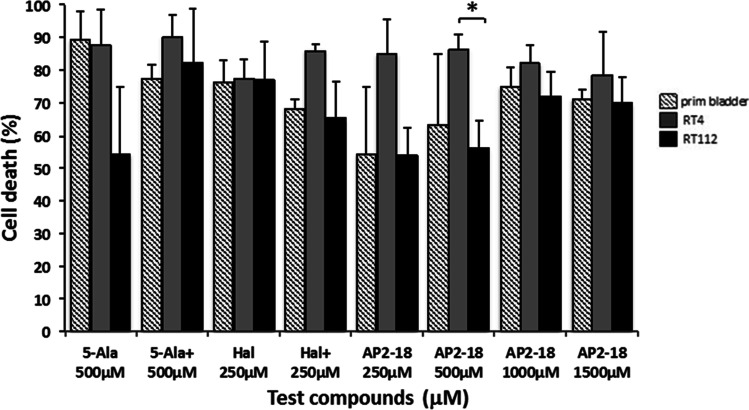
Mean percentage cell death recorded in normal bladder cells and RT-4 & RT-112 bladder cancer cells following PDT conducted at 6 h (λ = 635 nm; light dose = 30Jcm^−2^) as assessed by the neutral red assay normalized to a reference of untreated control cells. The cells were treated with ALA alone (5-Ala), ALA + CP94 (5-Ala +), HAL alone (Hal), HAL + CP94 (Hal +), or the composite drug AP2-18 in increasing concentrations (from 250 to 1500 µM). CP94 was added in equimolar concentrations. Any statistical significance (Welch two-sample t-test) detected was indicated as *p ≤ 0.05. Bars indicate the standard error of the mean

Confocal laser scanning microscopy (Leica TCS SP8 with HyD; Milton Keynes, UK) was undertaken on all three bladder cell types in a separate but complimentary series of experiments. Sterile coverslips were placed in a 6-well plate and coated with attachment factor (Sigma, UK). Fifty thousand cells in 2 ml media were seeded onto the coverslips and incubated (37 °C/5% CO_2_) overnight. Treatment was conducted as above before the cells were fixed. Growth media was removed, and 4% para-formaldehyde in PBS pH7 (PFA) added to just cover the cells for 10 min at room temperature. This was removed, and the coverslips washed twice for 5 min with PBS/0.2% triton to permeabilize the cells, followed by a further three washes with PBS to remove any traces of detergent/PFA. Antibody and subsequent incubation steps took place in a humidified chamber. Phalloidin 488 (Abcam, Cambridge, UK; diluted 1:1000 in PBS/0.2% triton for 1 h at room temperature and under light protection) and Hoechst 33,342 (Sigma, UK; final concentration of 1:10,000 for the last 15 min) stains were utilized to highlight f-actin and DNA respectively. Caspase-3 was also qualitatively visualized via immunohistochemistry conducted with anti-human cleaved caspase-3 and Alexa 546. Cells were blocked with 5% goat serum in PBS/0.2% triton for 1 h at room temperature. This was removed before incubation with rabbit anti-human cleaved caspase-3 (Cell Signaling Technology, London, UK; 1:400 in 5% goat serum in PBS/0.2% triton) overnight at 4˚C. The next day, the primary antibody was washed off thoroughly with PBS/0.2% triton and PBS before adding the secondary antibody (antibody goat anti rabbit Alexa546 (Invitrogen, ThermoFisher, Paisley, UK; 1:400 in PBS) and incubating for 4–6 h at room temperature. For counterstaining, phalloidin 488 and Hoechst 33,324 were added for the last 1 h and 15 min respectively, as described above. After the incubation steps, the coverslips were washed three times with PBS/0.2% triton and another three times with PBS before being mounted on microscope slides with ProLong Diamond Antifade (Invitrogen, ThermoFisher, Paisley, UK). For live cell images, 30,000 RT4 cells were seeded into a 35-mm diameter imaging µ-dish with a glass bottom (Ibidi, Graefelfing, Germany) and treated with 500 µM AP2-18 for 6 h (this concentration of AP2-18 was selected after considering the findings presented in Figs.1d–g and 2 in detail and concluding that the higher doses investigated did not convey additional benefit at this time point). Treatment media were replaced with RPMI/5% FBS/10% HEPES without phenol red. For single scan live cell imaging, the incubation box that houses the stage and microscope head was heated to 37˚C.

## Results

### Human bladder cell findings

Generation of standard curves demonstrated that PpIX fluorescence was linearly aligned to concentration (Fig. 1a–c). Near linear PpIX fluorescence was produced up to 6 h in all cases by AP2-18 in the RT-112 bladder cancer cells evaluated with ALA or HAL incubated with an equimolar amount of the iron chelator CP94 (Fig. 1d–f). The progression followed a similar gradient within each experimental group, and the fluctuation (indicated by the error bars) was relatively small. After 6 h, the fluorescence emissions diverged a little, as reflected in the higher standard errors presented. There were also indications with 1000 µM HAL + CP94 in particular that the accumulation of PpIX fluorescence was slightly slower after this time point. Statistical analysis indicated that fluorescence levels at 6 and 10 h were not significantly different from one another. Although the fluorescence intensities generally increased over time, they did not necessarily increase with increasing concentrations of the prodrug. RT-112 cells treated with 500 µM ALA + CP94 generated more PpIX than when treated with 1000 µM (Fig. 1d). The same observation was made when RT-112 cells were treated with HAL + CP94, where the highest fluorescence levels were reached with 250 µM (Fig. 1e). AP2-18 appeared more dose dependent in this cell type, although the highest individual measurement of PpIX fluorescence recorded was also with 500 µM (Fig. 1f). The differences in fluorescence emissions from 250 to 1000 µM were not statistically significant. PpIX fluorescence accumulated by the combinational prodrug AP2-18 was not dissimilar to that produced by ALA + CP94 or HAL + CP94 administered separately.

PpIX accumulated by all three bladder cell types with all eight treatment variations is presented (Fig. 1g). RT-4 cells’ fluorescence exceeded primary bladder and RT-112 cells with all the variations considered, up to four times in intensity and reaching 15-17A.U. (two sample Welch t-test, p-values 0.0050–0.0001). For RT-112 and primary bladder cells, fluorescence levels hardly reached 10A.U. Although CP94 addition to ALA or HAL did produce slightly elevated PpIX accumulation in all cell types, this was not statistically significant. PpIX accumulation was lowest in primary bladder cells treated with 250 or 500 µM AP2-18 (1.6 and 3.3A.U. respectively), which were both significantly lower than the higher concentrations of AP2-18 or ALA/HAL ± CP94. Once the AP2-18 concentration reached 1000 µM, the combinational drug was just as effective at accumulating PpIX in this cell type as all the other prodrug combinations tested and increasing to 1500 µM AP2-18 did not deliver further improvement. Although the arbitrary PpIX levels observed were much higher, the same basic trend was observed in the RT-4 cells, i.e., significantly lower PpIX accumulating with 250 or 500 µM AP2-18 than all the other experimental variations (with PpIX fluorescence doubling between these two concentrations) and all other groups only being marginally different and hence insignificant on statistical analysis (p > 0.05). The PpIX levels produced within the RT-112 cells were all reasonably similar to one another with no clear trends being identified.

The neutral red values of the cells that survived PDT (drug light interval = 6 h) were subtracted from the values of the treatment control cells (Fig. 2). The results therefore represent the level of cell death presented as a percentage. The variation observed between groups was less marked than when measuring PpIX fluorescence. Generally, RT-4 cells seemed to be more sensitive to PDT than RT-112 cells, which was in keeping with the fluorescence findings (Fig. 1g), although the differences in cytotoxicity were not found to be statistically different in all but one case (PDT cytotoxicity was significantly greater with 500 µM AP2-18 in RT-4 rather versus RT-112 cells (p < 0.05)). Cell death in primary bladder cells was on par with the rates of cell death observed in RT-112 cells, which was again in keeping with the previous PpIX measurements (Fig. 1g). The addition of CP94 to the individual prodrug incubations did not convey increased PDT cytotoxicity in these cells of bladder origin and with HAL appeared to reduce it in two of the three instances investigated albeit in a non-significant manner (Fig. 2).

### Human epithelial squamous carcinoma cell findings

**Fig. 3:**
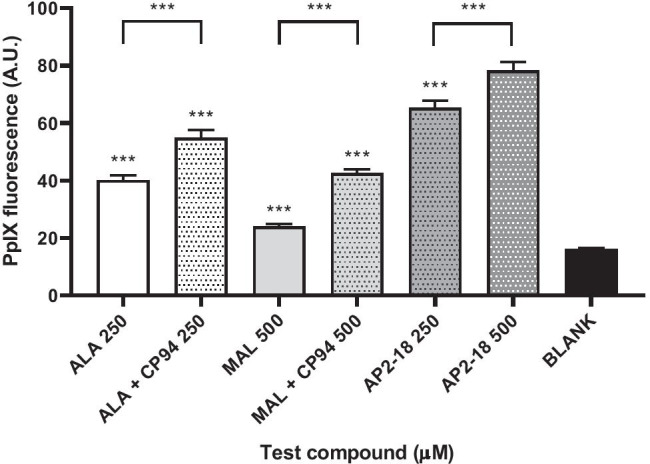
Mean PpIX fluorescence measured after 6-h incubation in primary epithelial squamous carcinoma cells (A431; n = 3). The cells were treated with ALA ± CP94, MAL ± CP94 or the composite drug AP2-18 in increasing concentrations (250 and 500 µM). CP94 was added in equimolar concentrations. Statistical significance (Welch two-sample t-test) between 500 µM AP2-18 and the other experimental groups and also between paired treatment conditions is indicated as ***p ≤ 0.001. Bars indicate the standard error of the mean

**Fig. 4:**
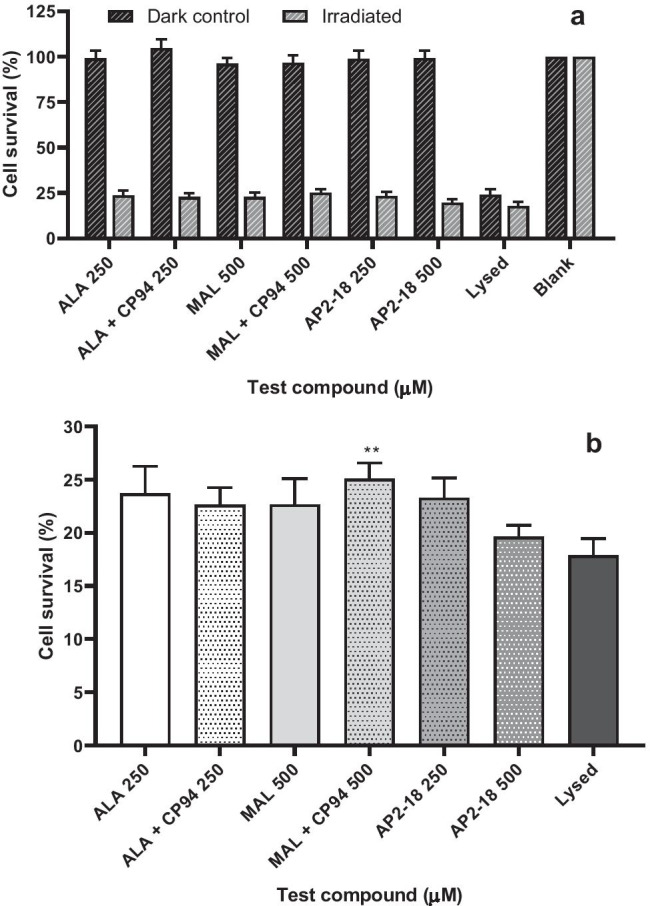
Mean percentage cell survival of primary epithelial squamous carcinoma cells (A431; n = 3) following PDT conducted at 6 h (λ = 635 nm; light dose = 37Jcm^−2^) as assessed by the neutral red assay. The cells were treated with ALA ± CP94, MAL ± CP94, or the composite drug AP2-18 in increasing concentrations. CP94 was added in equimolar concentrations. Panel **a** Non-irradiated dark control groups and irradiated PDT groups. Panel **b** Irradiated PDT data alone. Statistical significance (Welch two-sample t-test) between 500 µM AP2-18 and the other experimental groups on panel **b** is indicated as **p ≤ 0.01. Bars indicate the standard error of the mean

As the findings in the cells of bladder origin were unlike our previous observations of iron chelation, where we had always observed significant enhancement of PpIX accumulation within cells of dermatological origin even if this had not always been translated into increased cell death on irradiation [34, 35], further experimentation within human epithelial squamous carcinoma cells was undertaken. Initial PpIX monitoring experimentation (Fig. 3) clearly demonstrated that iron chelation significantly enhanced PpIX fluorescence accumulation in this cell type, with the highest dose of AP2-18 investigated (500 µM) significantly (p < 0.001) outperforming separate administration of CP94 with either ALA or MAL, even though the addition of separate CP94 had already significantly improved (p < 0.001) both ALA and MAL PpIX accumulation. The effectiveness of these PDT regimes in this cell type was also clearly demonstrated when compared with the corresponding non-irradiated dark control groups (Fig. 4a). Generally, the addition of an iron chelator either as separate CP94 or the combinational prodrug AP2-18 at the doses investigated did not convey any substantial additional cytotoxic effect over the already highly effective ALA alone or MAL alone PDT protocols (Fig. 4b). However, a visual trend towards greater cell kill (that reached statistical significance (p < 0.01) with MAL + CP94) was nonetheless evident with the higher dose of 500 µM AP2-18 employed with this 6-h drug light interval (Fig. 4b).

### Confocal microscopy findings

**Fig. 5:**
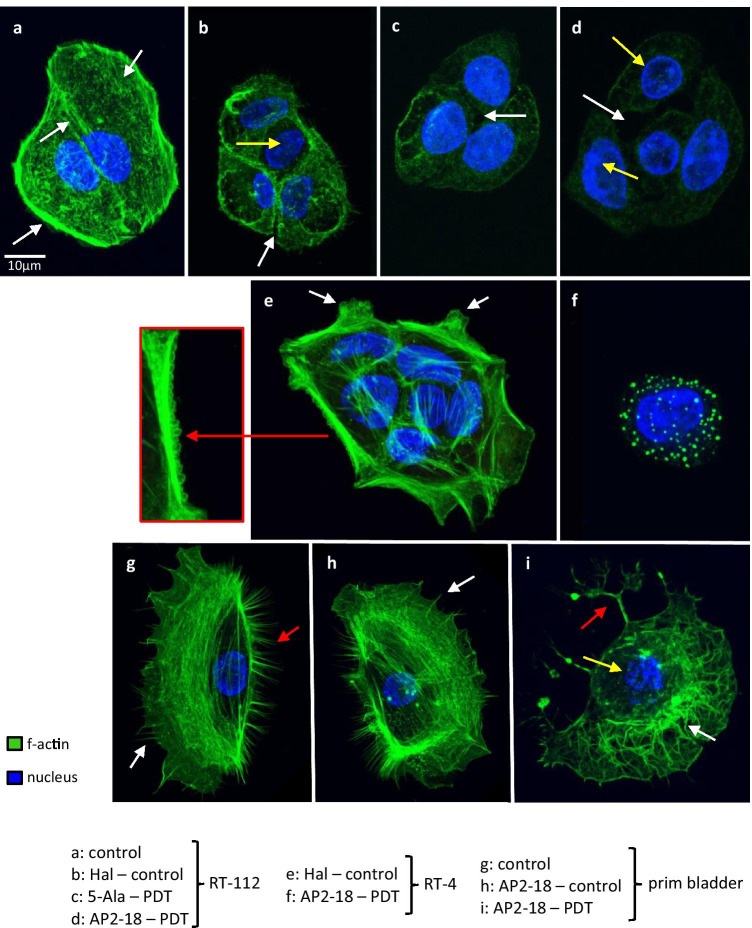

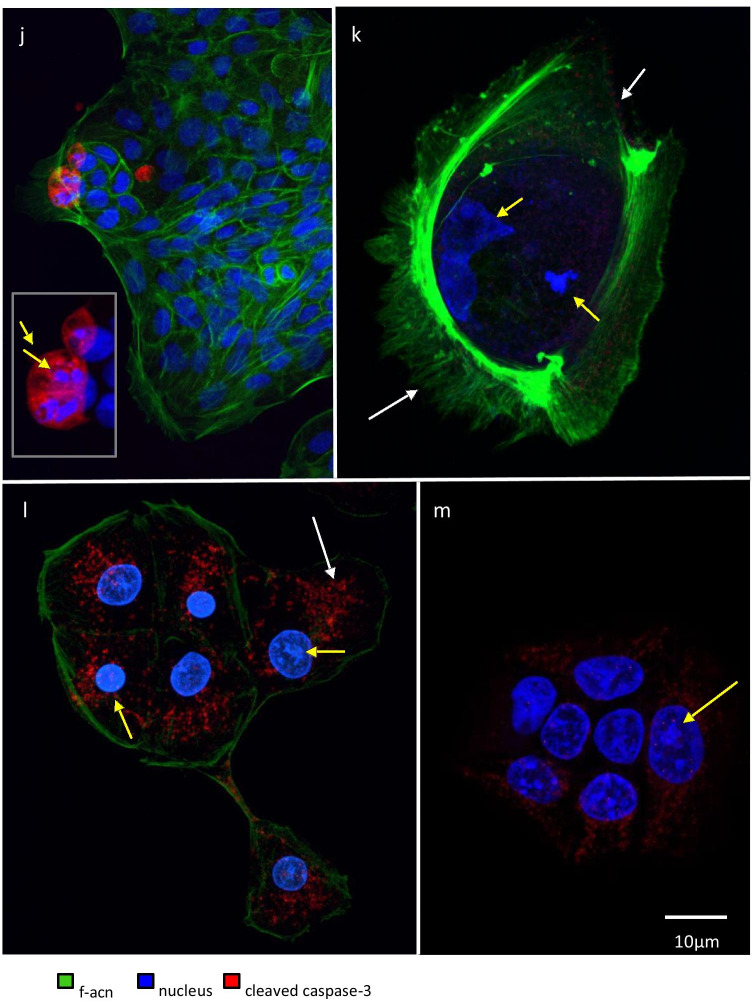
Confocal microscope images documenting f-actin depolymerization and cleaved caspase-3 in bladder cells after PDT. RT112, RT-4, and normal bladder cells seeded on cover slips were treated as detailed. The fixed cells were stained for nuclear DNA (Hoechst, blue) and f-actin (phalloidin 488, green) and underwent immunohistochemistry with anti-human cleaved caspase 3 and Alexa 546 (red). The confocal microscope images are representative for the controls, treatment with prodrug alone, or prodrug-PDT. Panels **a**–**i** show the dramatic decrease in f-actin and the loss of cytoskeletal structure elements. A detail of image **e** shows a row of characteristic f-actin blebbing on the cell membrane. Panels **j**–**m** document caspase-3 release. Panel **j** represents the untreated RT-4 control cells and the detail of a classic apoptotic cell, which were very low in numbers; **k** normal primary bladder cells after AP2-18-PDT; **l** RT-4 cancer cells after AP2-18-PDT and **m** RT-112 cells after ALA-PDT. All images were taken with the same settings [Leica SP8 with HyD]. The scale bars in panels **a**, **m** apply to all images, except the detail of panels **e, j**

Confocal microscopy of the cytoskeleton revealed a thick layer of f-actin enveloped the well attached untreated RT-112 control cells with delicate lamellipodia and filopodia protrusions (lower arrow Fig. 5a). Cell–cell contacts were intact and F-actin accumulated in the cytosol as rather short filaments (upper arrows Fig. 5a). Nuclei were evenly sized with a smooth surface. Despite being viable, RT-112 PDT HAL only control cells detached in large rafts from the bottom of the culture dishes, and confocal microscopy indicated a distinct lack of f-actin through reduced phalloidin 488 green fluorescence and emerging gaps in the cell–cell junctions (white arrow Fig. 5b). F-actin started to depolymerize, and structural elements associated with cell attachment disappeared. The nuclei were smaller and denser (yellow arrow Fig. 5b). PDT with ALA and AP2-18 (Fig. 5c, d respectively) further reduced phalloidin 488 fluorescence; f-actin was fragmented/degraded, and the cytoskeleton structural elements were missing with large gaps between cells (white arrows Fig. 5c, d). Blue spots of condensed deposits of nuclear DNA and a fine blue nuclear rim were also observed (yellow arrows Fig. 5d).

RT-4 cells and primary bladder cells stayed adherent in prodrug control groups (Fig. 5e, g, h respectively). Cell–cell junctions were mostly intact and the nuclei also appeared normal. Stabilizing actin stress fibers were present. Closer inspection revealed that the lamellipodia lacked filopodia (arrows Fig. 5e) and that the outer membrane had started blebbing (red arrow detail Fig. 5e). After AP2-18-PDT, the cytoskeleton of RT-4 cells was completely disintegrated (Fig. 5f). In a number of cells, small but dense spots of f-actin were observed orbiting the nucleus. Normal primary bladder cells had a large cell body with filopodia extending from beyond the lamellipodia on the leading edge (white arrow Fig. 5g) but also large bundles of filopodia on the rear end of the cell (red arrow Fig. 5g). After exposure to AP2-18 (Fig. 5h), the cell structure seemed unchanged with neither f-actin degradation nor signs of blebbing. Following PDT however, the leading edge lamellipodia gave way to tufts of filamentous f-actin protein winding through the cytosol (white arrow Fig. 5i); the rear filopodia bundles retracted (red arrow Fig. 5i) and the nucleus appeared fragmented (yellow arrow Fig. 5i).

Caspase-3 release (red) was also visualized before and after PDT (representative images Fig. 5j–m). The insert in Fig. 5j shows a classic apoptotic cell in the non-treated RT-4 control group. The shrunken cell body is full of cleaved caspase-3 and condensed chromatin. Primary bladder cells after 250 µM AP2-18 PDT showed no signs of caspase-3 release, but the nuclei were deformed/partially fragmented, and the f-actin lamellipodia interrupted (yellow and white arrows respectively Fig. 5k). The absence of caspase-3 and cytotoxicity findings suggest that PDT-induced cell death with these parameters resulted from primary necrosis in this normal cell type. F-actin was reduced to a thin layer that engulfed the RT-4 cells after 500 µM AP2-18 PDT, and lumps of DNA in the nuclei were visible (yellow arrows Fig. 5l). Tiny dots of red caspase-3 fluorescence scattered throughout the cytosol were also detected (white arrow Fig. 5l), which were not present in the control experiments. RT-112 cells after 500 µM ALA PDT looked similar to RT-4 cells with condensed nuclei and aggregated nuclear DNA being observed (yellow arrow Fig. 5m). Tiny dots of cytosolic caspase-3 were also present with increased intensity. RT-4 and RT-112 cells post-PDT therefore appeared in to be in the early stages of apoptosis.

## Discussion

Previously intercepting heme synthesis through iron chelator addition significantly enhanced PpIX accumulation/PDT cytotoxicity in human skin cancer and normal fibroblasts [26, 28, 29]. This was not replicated in the normal and malignant bladder cells investigated here (Figs. 1g and 2), although it was demonstrated once again within human skin cancer cells (Figs. 3 and 4). The levels of fluorescence recorded were more dependent on the bladder cell type than on the treatment variations (Fig. 1g). Doubling prodrug concentrations in RT-112 cells did not double PpIX accumulation (Fig. 1d–f). RT-4 cells produced significantly higher levels of PpIX than other cell types (Fig. 1g), possibly because of their metabolic rate. These higher levels did not necessarily translate into significantly higher PDT cytotoxicity (Fig. 2). Instead, even the modest level of RT-112 PpIX accumulation already reduced the cell viability produced on irradiation by ~ 66%. Average RT-4 cell death was 85%. So, although PpIX accumulation was increased by nearly 200%, RT-4 cell death was only increased by 20%, indicating that in different cell types, different levels of cell damage may be required to induce cell death.

Iron chelator addition, either as separate CP94 or prodrug linked as AP2-18, did not seem to have any consequential influence, either on PpIX fluorescence (Fig. 1g) or bladder cancer cell death following PDT (Fig. 2). This general lack of iron chelator enhancement probably resulted in the new combinational prodrug AP2-18 not conveying any significant additional benefit over and above the existing prodrugs in these bladder cells (unlike observed in dermatological cells [34, 35]), although it was equally as effective as separate co-administration.

The normal bladder cells were also as susceptible to PDT-induced cytotoxicity as the cancer cells (Fig. 2; ~ 71%) which would not be desirable for clinical photodetection/PDT application. To our knowledge, there is no record of investigating normal primary bladder cells for PDT, with little PDT effect on immortal UROtsa cells and N1 fibroblasts PDT-resistance being reported instead [37–39]. Here, ≤ 500 µM AP2-18 produced the least amount of PpIX in normal bladder cells (Fig. 1g) and correspondingly had the highest survival rate post-PDT (Fig. 2). As the same treatment parameters produced satisfactory RT-4 cancer cell cytotoxicity (Fig. 2), this may indicate that it is possible to improve the normal versus malignant contrast at this concentration.

In skin cancer cells, iron chelation consistently, significantly (p < 0.001) improved PpIX accumulation (Fig. 3) when separate CP94 was co-administered with ALA/MAL but was most pronounced when the combinational iron chelating PpIX prodrug AP2-18 was employed. 500 µM AP2-18 significantly increased (p < 0.001) PpIX accumulation above that produced by all other treatment regimens, corresponding with previous findings [34, 35]. Following PDT (Fig. 4), although the highest AP2-18 concentration produced a visual trend towards greater cytotoxicity, this only reached statistical significance (p < 0.01) when considered against MAL + CP94. Increasing the AP2-18 dose further may have resolved this but generally iron chelation works best when the treatment parameters are limited and thus sub-optimal [35]. The standard prodrug regimens utilized were already highly effective (Fig. 4) killing ≥ 75% of skin cancer cells without any augmentation being necessary. Future work should therefore continue to test AP2-18 within dermatological cells and explore more limited/sub-optimal standard prodrug treatment regimens with shorter drug-light intervals to try to replicate the clinical scenario at the base of thicker nodular BCC tumors where the current licensed protocol is currently producing inadequate results (without repeat treatments).

Battah et al. [32] found that ALA and CP94 directly linked via an ester function (like AP2-18) was the least efficient in a series of conjugates that they synthesized but was slightly less hydrophilic than ALA and so should be slightly better at entering cells [32]. Hydrophilicity could be decreased further by extending the ester bridge between ALA and CP94 but this would also increase molecular size. Many experimental factors differed (cell type; culture conditions; light source), making direct comparison difficult. No additional iron was employed here for instance as this is not required for effective tissue culture, and it is also hard to anticipate the optimal cytosolic iron levels required [40, 41]. Adding excess Fe^2+^ would selectively favor the chelating compound, while quenching PpIX accumulation in the other groups without necessarily reflecting the cells’ natural environment/capabilities.

It is also important to consider the potential deleterious effect that adding ever increasing amounts of iron chelating compounds could have on this complex series of biochemical events. Once the heme biosynthesis pathway is operating in an optimal manner for the maximal PDT effect possible, with very limited ferrocheletase activity left remaining to inhibit through additional iron chelation, further sequestering of cellular free iron could result in reduced ROS production and thus less cell death on irradiation, as iron can catalyze ROS generation following PpIX-induced PDT [41].

Palasuberniam et al. [42] have determined that ferrochelatase activity is an important determinant of tumor response to iron chelation with deferoxamine (DFO). Knock down cell types without ferrochelatase activity were more sensitive to ALA-PDT, but this alteration completely abolished the enhancement effect of DFO iron chelation. Although the ferrochelatase activity of the specific cell types employed here has not been established, it is postulated that this factor may at least in part explain the differing findings observed between the human cells of bladder and skin origin. A431 epithelial squamous carcinoma cells are known to express high levels of epidermal growth factor receptor (EGFR) [43] and lactase dehydrogenase (indicating elevated glycolic metabolism) [44] and strong expression of the PPARGC1A gene (suggesting mitochondrial biogenesis and oxidative phosphorylation) [44]. Although ALA-PDT has been investigated in RT-112 bladder cancer cells previously [45], we are not currently aware of their ferrochelatase activity, and so this would be an interesting avenue of future research.

Confocal microscopy imaging also revealed that prodrug-induced PpIX accumulation alone already destabilized the actin cytoskeleton in bladder cancer cells (particularly RT-112) prior to PDT (Fig. 5). Iron depletion (which could result from more PpIX being available than normal to bind Fe^2+^) can indirectly lead to f-actin depolymerization [46] and again would reduce the potential potentiating effects of iron chelation. The primary bladder cells, however, managed to maintain their cytoskeletal integrity under prodrug treatment. PDT further damaged the cytoskeleton in bladder cancer cells. Visualization of caspase-3 release post-PDT indicated that both bladder cancer cell types were in the early stages of apoptosis, whereas absence of caspase-3 in normal bladder cells suggested cell death from primary necrosis.

## Conclusion

Although all tested prodrugs raised PpIX levels significantly in treated cells and caused considerable cell death after PDT, none of the prodrugs ± CP94 iron chelation or the combined conjugate AP2-18 stood out as an optimal treatment regime in the bladder cells investigated. This is in direct contrast to the findings with human skin cancer cells, where both separate CP94 iron chelating supplementation to standard prodrug administration as well as the novel combinational iron chelating PpIX-prodrug AP2-18, conveyed significant advantage particularly in respect to PpIX accumulation. It is therefore concluded that the mechanism of action of PpIX-induced PDT is both complex and highly cell type dependent, and so potential enhancement techniques such as iron chelation cannot be universally applied without thorough prior investigation. These findings support further investigation of iron chelator enhancement of PpIX-induced PDT for dermatological applications, however, not bladder photodetection/PDT.
